# Green Synthesis of Carbon Dots and Their Integration into Nylon-11 Nanofibers for Enhanced Mechanical Strength and Biocompatibility

**DOI:** 10.3390/nano12193347

**Published:** 2022-09-26

**Authors:** Xu Chen, Ying Qin, Xinru Song, He Li, Yue Yang, Jiazhuang Guo, Tingting Cui, Jiafei Yu, Cai-Feng Wang, Su Chen

**Affiliations:** 1 State Key Laboratory of Materials-Oriented Chemical Engineering, College of Chemical Engineering, Nanjing Tech University, 5 Xin Mofan Road, Nanjing 210009, China; 2 Department of General Surgery, The Affiliated Jiangning Hospital of Nanjing Medical University, Nanjing 211100, China

**Keywords:** carbon dots, green synthesis, Nylon-11, nanofiber, mechanical properties, biocompatibility

## Abstract

Carbon dots (CDs) have been extensively explored to show good optical features, low toxicity, and good biocompatibility. Herein, we report the new synthesis of forsythia-derived CDs (F-CDs) and their incorporation into Nylon-11 nanofibers for improved mechanical properties and biocompatibility. F-CDs are prepared from a Chinese herb forsythia via a magnetic hyperthermia method in 90 s without the use of any organic solvents. The as-prepared F-CDs with rich surface functional groups can be well embedded into Nylon-11 nanofibers via electrospinning, providing Nylon-11/F-CD nanofiber mats with remarkably enhanced mechanical properties. With the incorporation of F-CDs at 10 wt% into the Nylon-11 nanofiber mats, the tensile strength increases from 7.5 to 16.6 MPa, and the elongation ratio at break increases from 39% to 125%. Moreover, the Nylon-11/F-CD nanofiber mats exhibit excellent cytocompatibility towards L929 fibroblast cells with cell viability of 96%. These findings may guide the development of various CD-embedded nanofiber mats with good mechanical properties and biocompatibility potentially useful for biomedical applications, such as tissue engineering scaffolds or wound dressing.

## 1. Introduction

Carbon dots (CDs) have received widespread attention worldwide since their first discovery in 2004 [1], owing to their favorable attributes such as multicolored fluorescence, excellent photostability, high water solubility, low toxicity, and good biocompatibility [2,3,4,5]. Such nanomaterials show various potentials, including cell labeling [6], bioimaging [7], fingerprint detection [8], anti-counterfeiting [9], luminescent solar concentrators [10], and flame retardancy [11]. Up until now, many attempts have been made in the preparation of CDs to discover various natural precursors, such as leaves [12], fruits [13], eggs [14], and turtle shells [15], and also to develop diverse synthetic routes, such as the hydrothermal method [16], the microwave method [17], the chemical oxidation method [18], or the arc discharge method [19]. Specifically, we previously developed a magnetic hyperthermia method allowing for the rapid and large-scale production of CDs from citrate and urea [20]. At present, the synthesis of CDs using a facile scale-up method and low-cost green materials as precursors is still of great interest.

Meanwhile, electrospinning fiber mats are extensively applied in research on tissue engineering and drug delivery owing to the advantages of a large specific surface area, high porosity, suitable cell adhesion, and a high drug-carrying capacity [21,22]. Electrospinning fiber mats made from natural polymers or biocompatible polymers meet most of these requirements. However, the key weakness of natural polymer materials such as collagen [23], chitosan [24], gelatin [25], and sericin [26] is their poor mechanical strength. As such, despite their various advantages, the mechanical strength of fiber mats remains to be enhanced to fulfill their widespread application as scaffolds in tissue engineering. Consequently, it is essential to prepare fiber mats with high mechanical properties and good biocompatibility.

**Figure 1:**
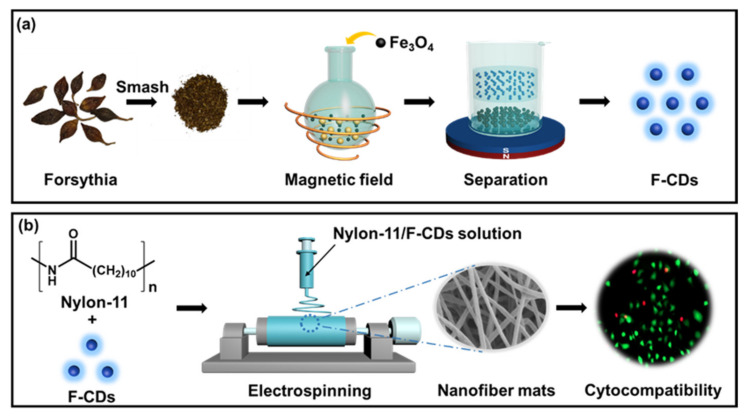
(**a**) Schematic diagram of the operating procedure for the synthesis of F-CDs by the magnetic hyperthermia method. (**b**) Schematic diagram of the preparation of biocompatible nanofiber mats via electrospinning.

This paper presents the rapid green synthesis of forsythia-derived CDs (F-CDs) as well as their integration into Nylon-11 nanofiber mats for enhanced mechanical strength and biocompatibility. As a vine, forsythia is a well-known herbal remedy with antibacterial and antiviral properties beneficial for treating inflammation, fever, and ulcers [27]. Forsythia leaves and fruits are rich in lignans, which have functional groups of alcoholic hydroxyl, carboxyl, and methoxy groups, often facilitating the generation of fluorescent CDs [28,29]. Nylon-11 is derived from castor oil cleavage products, a fully bio-based polymer with recyclable practicality. Electrospinning Nylon-11 nanofiber mats have demonstrated a tendency for application in cell culture [30]. Herein, with the use of forsythia as the green precursor, the rapid preparation of CDs was achieved using the magnetic hyperthermia method, where forsythia could be rapidly converted to F-CDs within 90 s (Figure 1a). Utilizing the advantages of CDs with rich surface functionalization and excellent biocompatibility properties, F-CDs were embedded into Nylon-11 nanofiber mats by electrospinning to give enhanced mechanical properties and good biocompatibility to Nylon-11 nanofiber mats (Figure 1b). With the incorporation of F-CDs at 10 wt%, the Nylon-11/F-CD nanofiber mats have 2.2 and 3.2 times higher tensile strength and elongation than the pure Nylon-11 nanofiber mats, respectively. Therefore, this work contributes a facile approach to preparing nanofiber mats with versatile properties, including high mechanical strength and excellent biocompatibility, which are promising to be applied in biomedical fields, such as artificial tissue scaffolds or wound dressing.

## 2. Materials and Methods

### 2.1. Materials

All materials were used as received. Dried forsythia fruits (a kind of Chinese herb, also named *Fructus Forsythiae*) were purchased from a local herbal market. Nylon-11 pellets, 1,1,1,3,3,3-hexafluoro-2-propanol (HFIP 99%), and iron oxide nanoparticles (Fe_3_O_4_ nanoparticles with a mean size of ca. 20 nm) were purchased from Sinopharm Chemical Reagent Co., Ltd. (Shanghai, China). Pure water was purchased from Hangzhou Wahaha Group Co., Ltd., Hangzhou, China. A live/dead animal cell viability assay kit (KGAF001) was purchased from Nanjing Keygen Biotechnology Co., Ltd., Nanjing, China. *Escherichia coli* (*E. coli*) and *Staphylococcus aureus* (*S. aureus*) were cultured on a plate medium (Hopebiol Biotech Co., Ltd., Qingdao, China).

### 2.2. Preparation of F-CDs by Magnetic Hyperthermia Method

First, the forsythia fruits were cleaned thoroughly with pure water, dried at 50 °C in an oven, and then crushed into powder by a ball mill. Next, 50 g of the forsythia powder and 5 g of the Fe_3_O_4_ nanoparticles were placed in a 100 mL round-bottom flask and stirred evenly. Then the round-bottom flask was placed in the induction coil of a magnetothermal reactor (AtecD-20/380, Shanghai Bamac Electric Technology Co., Ltd., Shanghai, China) under 100 kHz and 13 kW, and an infrared thermometer (optris CTlaser LT, OPTCTL3MLCF, Berlin, Germany) was used to monitor the temperature during the reaction. Specifically, the Fe_3_O_4_ nanoparticles were used as the heat source to heat the precursors in an induction magnetic field, allowing the temperature of the whole reaction system to rapidly reach ca. 230 °C within 5 s. After 90 s, the resultant products were cooled and collected carefully. Subsequently, the samples were dispersed in a beaker with pure water and ultrasonicated for an hour. A magnet was placed under the bottom of the beaker for 10 min to remove the Fe_3_O_4_ nanoparticles. Then, the suspension was filtered by a filtering membrane (pore size: 0.22 μm), and then dialyzed in 1000 mL of dialysate (pure water) for 2 days with the use of a dialysis bag (molecular weight cut-off: 1000 Da), by changing the dialysate twice a day. Finally, the obtained F-CDs in the dialysis bag were dried and collected as F-CD powders for further characterization and application.

### 2.3. Preparation of Nylon-11/F-CDs Nanofiber Mats

The Nylon-11/F-CD nanofiber mats were prepared by a microfluidic electrospinning machine (JNS-MS-05-ME01, Nanjing Janus New-Materials Co., Ltd., Nanjing, China). The details are as follows: 1.5 g of Nylon-11 was dissolved in 13.5 g of HFIP and stirred for 6 h at room temperature until it was dissolved completely. Then, a certain mass of F-CD powders was added to obtain a mixed solution of Nylon-11/F-CDs. Specifically, we prepared five sets of Nylon-11/F-CD co-blended solutions with mass fractions of 0 wt%, 1 wt%, 2 wt%, 5 wt%, and 10 wt% for F-CDs, respectively. The solutions were sonicated for 5 min to remove air bubbles. The spinning solution was placed in a 5 mL plastic syringe fitted on a microfluidic injection pump. Electrospinning was performed at a constant injection rate of 1 mL/h with a voltage of 20 KV and a distance of 10 cm between the aluminum collector and the tip of the nozzle. A spinning needle with an inner diameter of 0.3 mm was used. The spinning process was carried out at a temperature of 25 °C and relative humidity of 55%. Finally, the Nylon-11/F-CD nanofiber mats were obtained, which were dried at room temperature for 1 day to completely remove the residual solvents.

### 2.4. Antibacterial Ability of Nylon-11/F-CDs Nanofiber Mats

The Gram-positive bacteria *S. aureus* and Gram-negative bacteria *E. coli* were used to investigate the antibacterial activities. The precursor solution was sterilized by 200 nm syringe filters. First, the nanofiber mats were put into the 96-well plate and sterilized by UV irradiation for 1 h. PBS buffer (pH 7.4) was added to wash off the unreacted materials three times. Subsequently, 100 µL of bacteria solution (104 CFU mL^−1^) was poured into each well, and an empty well was used as a control. The bacteria were incubated in a 96-well plate for 24 h at 37 °C, after which the suspension was taken out and mixed with 100 µL of Luria–Bertani (LB) medium, and seeded on a Nutrient Agar Plate to continue to incubate for 48 h. Then they were collected using PBS. The collected solution was centrifuged at 8000 rpm for 1 min at 25 °C, the supernatant was discarded, and 2.5% glutaraldehyde was added and mixed well. Then the solution was fixed at 4 °C for 12 h. Finally, the sample was treated with ethanol gradient dehydration, and then resuspended with 300 μL absolute ethanol. A suspension (10 μL) was used to put the solution on clean glass slides, dry it, and observe it with an electron microscope.

### 2.5. Cell Viability and Biocompatibility of Nylon-11/F-CDs Nanofiber Mats

The biocompatibility of the Nylon-11/F-CD nanofiber mats was tested with live/dead cell staining. Prior to cell testing, the nanofiber mat was put into a 24-well plate and sterilized using UV irradiation. L-929 fibroblast cells were cultured with 10% fetal bovine serum at 37 °C and 5% CO_2_, and then adjusted to a cell concentration of 5 × 10^4^ cells/well with the use of 10% fetal bovine serum. Subsequently, 1 mL of cell suspension was added to the 24-well plate. After co-culturing for 3 days, the live and dead cells were stained with Calcein-AM solution and PI solution, respectively. The stained cells were observed using a fluorescence microscope and photographed.

### 2.6. Characterizations

Transmission electron microscopy (TEM) images of the F-CDs were taken by an FEI Tecnai G2 F20 electron microscope. X-ray diffraction (XRD) patterns of the F-CDs, Nylon-11 nanofiber mats, and Nylon-11/F-CD nanofiber mats were revealed on a Bruker AXS D8 ADVANCE X-ray diffractometer. The Fourier transform infrared (FT-IR) spectra of the F-CDs, Nylon-11 nanofiber mats, and Nylon-11/F-CD nanofiber mats were recorded on a Nicolet 6700 FT-IR spectrometer. X-ray photoelectron spectroscopy (XPS) of the F-CDs was performed with a Thermo ESCAIAB 250XI XPS system. A PerkinElmer Lambda 900 UV–vis spectrophotometer was employed to measure the ultraviolet–visible (UV–vis) absorption spectrum of the F-CDs. The fluorescence properties of the F-CDs, including the emission spectra and lifetime curves, were measured using an Edinburgh FLS 980 spectrometer. Scanning electron microscopy (SEM) images were taken using a Hitachi S-4800 scanning electron microscope. Fluorescence images of the Nylon-11/F-CD nanofiber mats were taken using a Leica TCS SP5 laser scanning confocal microscope (Leica Company, Wetzlar, Germany).Leica SP5 The mechanical properties were investigated using a SANS CMT6203 testing machine. Fluorescence microscope images of the live/dead staining L-929 cells were recorded using an Opera Phenix (PerkinElmer Inc., Seer Green, UK).

## 3. Results and Discussions

### 3.1. Synthesis and Characterizations of F-CDs

**Figure 2:**
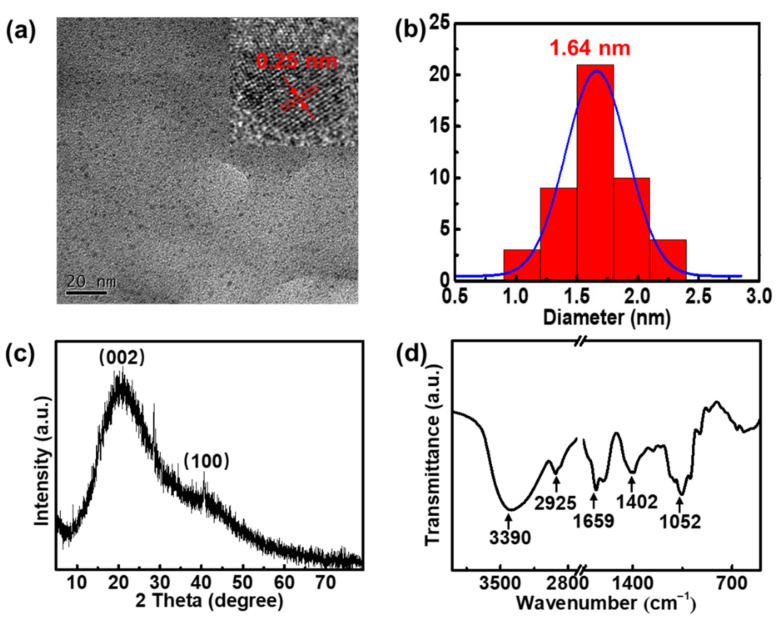
(**a**) TEM image of F-CDs. Inset: HRTEM image of a single dot. (**b**) Diameter distribution of F-CDs; (**c**) XRD pattern of F-CDs. (**d**) FT-IR spectrum of F-CDs.

In this study, the rapid synthesis of CDs was achieved with magnetic hyperthermia treatment of forsythia for 90 s (Figure 1a). The carbon source, forsythia, is a natural vine that is non-toxic, renewable, and environmentally friendly. The magnetic hyperthermia preparation procedure is simple and rapid, whilst circumventing the use of organic solvents. Therefore, forsythia-derived CDs (F-CDs) were prepared in a relatively green way. We investigated the morphology and general appearance of the F-CDs. Figure 2a shows a TEM image of the F-CDs, from which we can see that the F-CDs were well-dispersed nanoparticles. Then, we studied the diameter distribution of the F-CDs, as shown in Figure 2b, showing that the particle sizes mostly ranged from 1.05 to 2.25 nm, with a mean size of 1.64 nm. Furthermore, the high-resolution TEM (HRTEM) image demonstrates the high crystallinity of the F-CDs with a lattice spacing of 0.25 nm, which is consistent with that of the (100) plane of graphene [31], reflecting the graphitic nature of the F-CDs (inset in Figure 2a). For the XRD pattern of the F-CDs, there were two peaks at 2θ = 20.6° and 40.7° (Figure 2c), corresponding to the graphite structure of CDs [32]. Figure 2d displays the FT-IR spectra of the F-CDs. The broad band ranged from 3500 to 3000 cm^−1^, with a peak at 3390 cm^−1^, which may belong to O–H and N–H stretching vibrations. The absorption peak at 2925 cm^−1^ was due to the presence of C–H. Strong peaks at 1659 and 1402 cm^−1^ could be assigned to C=O and C=C/C–N stretching vibrations, respectively. Additionally, the peak at around 1052 cm^−1^ corresponds to C–O vibrations [33].

**Figure 3:**
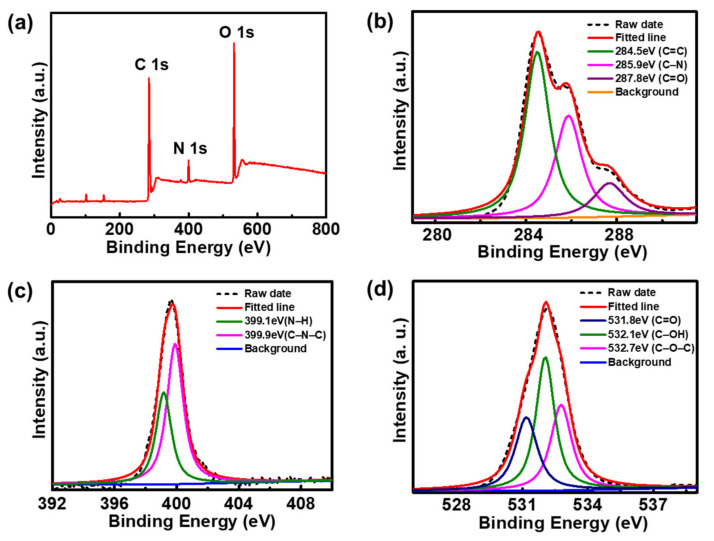
(**a**) XPS full survey spectra of F-CDs, and the corresponding (**b**) C 1s, (**c**) N 1s, and (**d**) O 1s high-resolution XPS spectra of F-CDs.

XPS measurements were carried out to further research the surface functional groups as well as the elemental composition of the F-CDs (Figure 3). The XPS full survey spectrum of the F-CDs reveals three typical peaks at 285.1, 400.2, and 532.1 eV, indicating the existence of C, N, and O elements in the F-CDs (Figure 3a). Three bands, at 284.5, 285.9, and 287.8 eV, were observed in the high-resolution XPS spectrum of C 1s (Figure 3b), which can be assigned to the C=C, C–N, and C=O groups, respectively [34]. Figure 3c shows the XPS spectrum of N 1s, which presents two peaks at 399.1 and 399.9 eV, belonging to the N–H and C–N–C groups, respectively [9,35]. The XPS spectrum of O 1s displays three bands at 531.8, 532.1, and 532.7 eV (Figure 3d), corresponding to the C=O, C–OH, and C–O–C groups, respectively [9]. The functional groups identified by XPS and FT-IR are consistent. The above results indicate that the F-CDs contained a variety of surface functional groups (hydroxyl, carbonyl, amino, etc.). These polar functional groups endow F-CDs with excellent water solubility and facilitate their further modification and application.

### 3.2. Optical Characteristics of F-CDs

**Figure 4:**
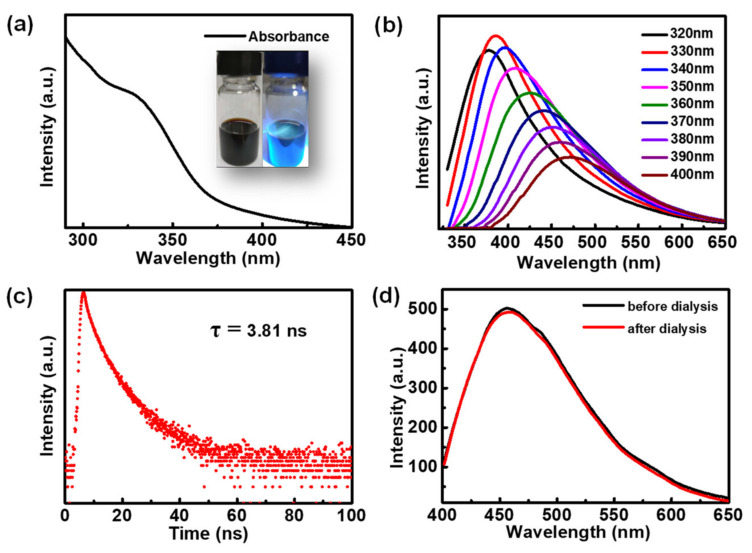
(**a**) UV–vis absorption spectrum of an aqueous solution of F-CDs. The inset shows digital pictures of an aqueous solution of F-CDs taken under daylight and an ultraviolet light, respectively. (**b**) PL emission spectra of F-CDs measured under different excitation wavelengths starting from 320 to 400 nm. (**c**) PL decay curve of F-CDs obtained at emission wavelength of 385 nm. (**d**) PL emission spectra of F-CDs during the pre- and post-dialysis periods.

The optical characteristics of the F-CDs were studied. The UV–Vis absorption spectrum of the F-CDs dispersed in water reveals absorption at 325 nm (Figure 4a), which could be ascribed to the n–π* transition of the C=O bond [36]. The insets in Figure 4a reflect the dark brown color of the F-CD suspension under daylight, while it had a blue fluorescence color under the 365 nm UV light. The as-prepared F-CDs demonstrated a typical excitation-dependent fluorescence (Figure 4b). When the excitation wavelength increased from 320 to 400 nm, the maximum photoluminescence (PL) emission wavelength of the F-CDs gradually shifted from 375 to 480 nm. Consequently, we found that F-CDs have an optimal excitation wavelength of 330 nm and an optimal PL emission wavelength of 385 nm. The fluorescence lifetime of F-CDs was studied using time-resolved PL decay measurements to give a lifetime of 3.81 ns monitored at 385 nm (Figure 4c). Furthermore, we investigated the fluorescence stability of the F-CDs during post-treatment. As shown in Figure 4d, there was no significant change in the fluorescence of F-CDs during the pre- and post-dialysis periods.

### 3.3. Microstructure and Reinforcement Mechanism of Nanofibers

**Figure 5:**
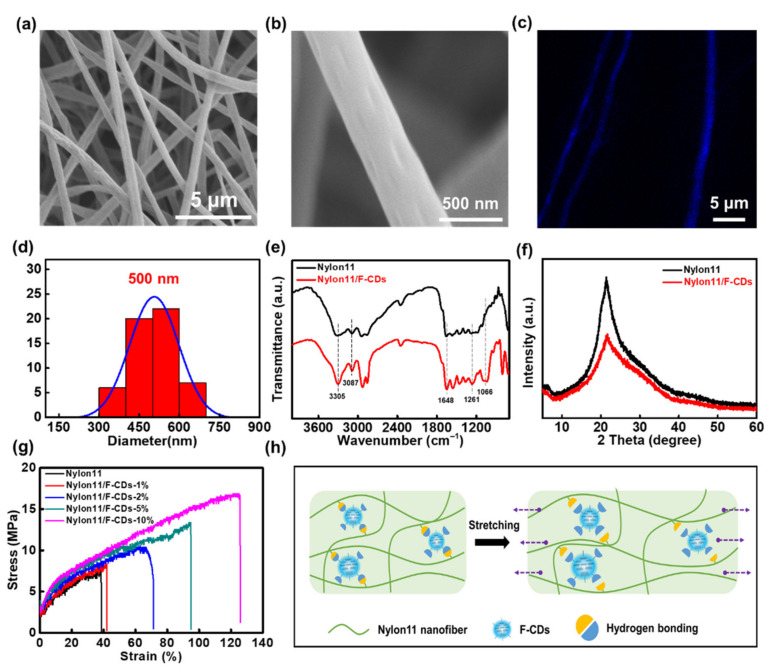
(**a**,**b**) SEM images of Nylon-11/F-CD nanofiber mats. (**c**) Fluorescent image of Nylon11/F-CD nanofiber mats acquired by a laser scanning confocal microscope. (**d**) Diameter distribution of Nylon-11/F-CD nanofiber mats. (**e**) FT-IR and (**f**) XRD spectra of Nylon-11 and Nylon-11/F-CD nanofiber mats. (**g**) Strain–stress curves of Nylon-11/F-CD nanofiber mats with different concentrations. (**h**) Schematic diagram of the mechanical enhancement mechanism of Nylon-11/F-CD nanofiber mats.

Interestingly, F-CDs with abundant surface functional groups could be well incorporated into Nylon-11 nanofibers via microfluidic electrospinning to obtain Nylon-11/F-CDs hybrid nanofiber mats with enhanced mechanical strength and biocompatibility. The typical microstructure of the as-prepared Nylon-11/F-CD nanofiber mats is well illustrated by the SEM images, as shown in Figure 5a,b. We can clearly see that the nanofiber mat is composed of uniform fibers with an average diameter of 500 nm (Figure 5c), which was comparable to the previously reported nanofiber diameters [37]. To confirm the good dispersion of the F-CDs in the Nylon-11 fibers, a fluorescence confocal microscopy image of a Nylon-11/F-CD nanofiber mat was measured, which exhibits a bright-blue fluorescence color along the whole fiber (Figure 5d). This feature suggests the uniform dispersion of Nylon11/F-CDs fiber without significant phase separation between the nanoparticles and the polymer matrix. The FT-IR spectra of the Nylon-11/F-CD nanofiber mats show N–H, =C–H, C=O, and C–O–C vibrations at 3305, 3087, 1648, and 1066 cm^−1^, respectively (Figure 5e). The rich surface functional groups on the F-CDs could result in good compatibility as well as hydrogen bonding between the F-CDs and the Nylon-11. Figure 5f shows the XRD spectra of the Nylon-11 and the Nylon-11/F-CD nanofiber mats. A broad reflection can be observed at 2θ = 21.6°, which indicates a weak and ordered hexagonal γ crystalline structure of Nylon-11 [38]. This sub-stable structure may have been formed due to the effect of the two simultaneous events of the rapid evaporation of the solvent and the lengthening of the polymer chains during the electrospinning, and it is more favorable for its application in tissue engineering [37].

Significantly, the Nylon-11/F-CD nanofiber mats show improved mechanical properties in terms of tensile strength and elongation. Figure 5g demonstrates the mechanical properties of the nanofiber mats fabricated at different concentrations of F-CDs. We can see that the stress–strain curves of the Nylon-11/F-CD nanofiber mats could be regulated by simply changing the F-CD concentration. Pure Nylon-11 nanofiber mats show an elongation ratio at a break of 39% at a stress intensity of 7.5 MPa. Whereas the increase in the concentration of F-CDs caused enhanced mechanical strength of the hybrid nanofiber mats. It is noteworthy that when the F-CD concentration was 10 wt%, the tensile strength was 16.6 MPa and the elongation was 125%. Therefore, with the incorporation of F-CDs at 10 wt% into the nanofiber mats, the tensile strength and elongation ratio at the break increased by 2.2 and 3.2 times, respectively. Due to the richness of the functional groups on the surface of CDs, CDs can easily interact with the polymer matrix through intermolecular interactions, for instance, hydrogen bonding [39]. As shown in Figure 5h, the presence of these hydrogen bonds not only provides abundant binding sites, bearing cross-sectional mechanical stresses during the stretching process, but also can serve as sacrificial units, consuming a large quantity of energy. Thus, the F-CD-embedded Nylon-11 nanofiber mats show enhanced mechanical properties.

### 3.4. Antibacterial Activity of Nanofibers

**Figure 6:**
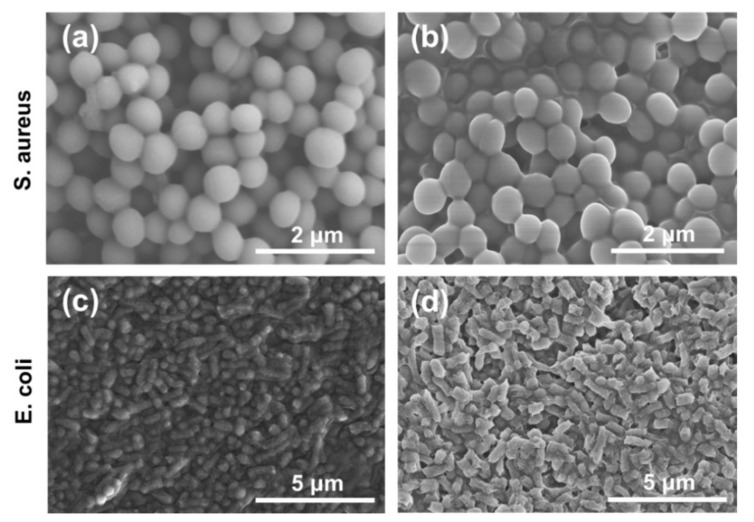
SEM images of S. aureus cells at 0 h (**a**) and 2.5 h (**b**), and *E. coli* cells at 0 h (**c**) and 2.5 h (**d**) co-cultivation with Nylon-11/F-CD nanofiber mats.

To investigate the feasibility of the Nylon-11/F-CD nanofiber mats for biomedical applications, preliminary antibacterial tests of these nanofiber mats were conducted. Specifically, the as-prepared Nylon11/F-CD nanofiber mats were co-cultured with two types of bacteria, *S. aureus* and *E. coli*, respectively. The morphology images of the bacteria at the times of 0 h, 2.5 h, and 24 h co-cultivation with the Nylon-11/F-CD nanofiber mats are shown in Figure 6 and Figure S1. Without the Nylon-11/F-CD nanofiber mats, the *S. aureus* cells and *E. coli* cells kept their structure and morphology, with a smooth surface (Figure 6a,c). However, upon co-culturing with the Nylon-11/F-CD nanofiber mats (Figure 6b,d and Figure S1), the cell membranes of the bacteria were disrupted, resulting in the deformation of the bacteria, the adhesions between the bacteria, as well as the flowing out of the internal lysate. As a well-known herbal remedy, forsythia shows antibacterial, antiviral, anti-inflammatory, and wound-healing activities [27]. The results suggest that the Nylon-11 nanofiber mats loaded with F-CDs derived from forsythia could also inhibit bacterial proliferation to some extent, while further insight into the antibacterial activity of F-CDs in future work is needed.

### 3.5. Cytotoxicity and Biocompatibility of Nanofibers

**Figure 7:**
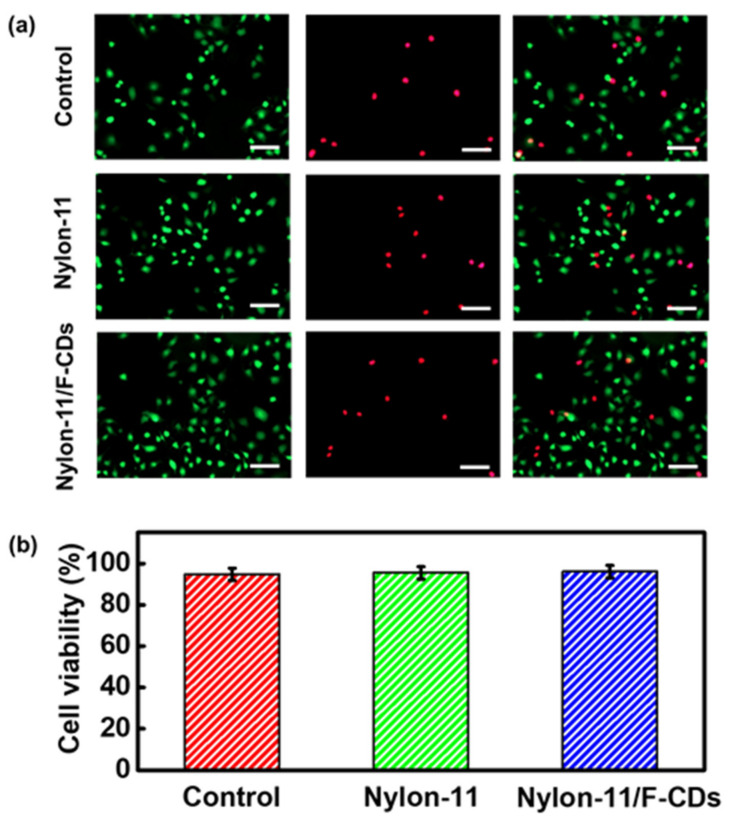
(**a**) Fluorescence microscope images of control, Nylon-11, Nylon-11/F-CDs with encapsulation of live/dead staining L-929 cells after culturing for 3 days. Red and green colors represent dead and live cells, respectively. Scale bar = 100 μm. (**b**) Relative survival of L-929 cells after culturing for 3 days for control, Nylon-11, and Nylon11/F-CD groups.

We further investigated the cytocompatibility of the Nylon-11/F-CD nanofiber mats. A live/dead cell staining assay was performed to assess the cytotoxicity of the Nylon-11 and Nylon-11/F-CD nanofiber mats in vitro. L929 fibroblast cells were seeded and cultivated for 3 days for the Nylon-11 and Nylon-11/F-CDs groups. We compared the cell viability of the different groups with a blank experiment, as shown in Figure 7. After culturing for 3 days, all three groups showed obvious cell proliferation, with cell viability of 96% for the Nylon-11/F-CD nanofiber mats and of 95% for the other two groups. The results suggest excellent cytocompatibility of the Nylon-11/F-CD nanofiber mats and non-toxicity of the F-CDs. Therefore, the integration of F-CDs into Nylon-11 nanofibers maintains good biocompatibility of the final nanocomposite product, whilst endowing the nanofibers with highly enhanced mechanical properties. Nylon-11/F-CD nanofiber mats have the benefits of non-toxicity, good biocompatibility, and excellent mechanical strength, which are expected to find potential in biomedical fields, including artificial tissue scaffolds and wound dressing.

## 4. Conclusions

We demonstrated the green synthesis of F-CDs and the preparation of Nylon-11/F-CD nanofiber mats with enhanced mechanical properties and good biocompatibility. F-CDs were synthesized using a magnetothermal method, where forsythia was rapidly converted into F-CDs within 90 s. Nylon-11/F-CD nanofiber mats were prepared by electrospinning. The mechanical properties of the Nylon-11/F-CD nanofiber mats were significantly improved. With the incorporation of F-CDs at 10 wt% into the Nylon-11 nanofiber mats, the tensile strength and the elongation ratio at the break were increased by 2.2 and 3.2 times, respectively, reaching 16.6 MPa and 125%, respectively. The in vitro cytotoxicity test on the L-929 fibroblasts show that the Nylon-11/F-CD nanofiber mats had good cytocompatibility. The results indicate that the prepared Nylon-11/F-CD nanofiber mats might find potential in wound dressing and tissue engineering applications.

## Data Availability

Not applicable.

## Supplementary Materials

The following supporting information can be downloaded at: https://www.mdpi.com/article/10.3390/nano12193347/s1, Figure S1: SEM images of *E. coli* cells at 24 h co-cultivation with Nylon-11/F-CDs nanofiber mats.
