# Supplementary material for: Green Synthesis of Carbon Dots and Their Integration into Nylon-11 Nanofibers for Enhanced Mechanical Strength and Biocompatibility
Source: Nanomaterials (Basel). 2022 Sep 26;12(19):3347. doi: 10.3390/nano12193347 (PMC9565341; doi:10.3390/nano12193347)
Supplement: Supplementary file 1 [file nanomaterials-12-03347-s001.zip › nanomaterials-1897068-supplementary.pdf]

Supplementary Materials

Green Synthesis of Carbon Dots and Their Integration into Nylon-11 Nanofibers for Enhanced Mechanical Strength and Biocompatibility

Xu Chen, Ying Qin, Xinru Song, He Li, Yue Yang, Jiazhuang Guo, Tingting Cui, Jiafei Yu *, Cai-Feng Wang * and Su Chen

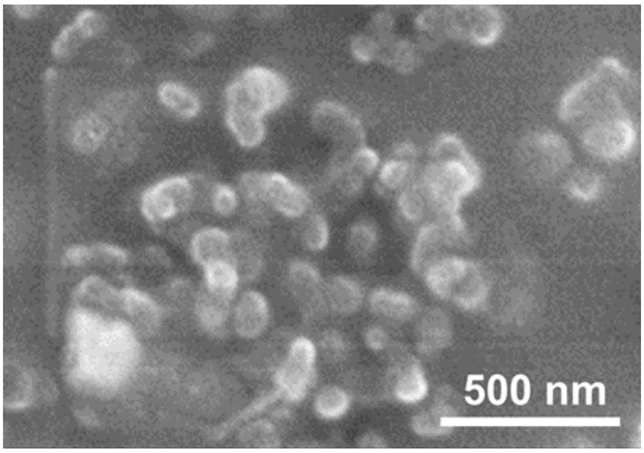

Figure S1. SEM images of *E. coli* cells at 24 h co-cultivation with Nylon-11/F-CDs nanofiber mats.
